# Women in Academic Psychiatry: A Mind to Succeed

**DOI:** 10.1192/pb.bp.116.055913

**Published:** 2017-10

**Authors:** Linda Gask

As a female academic psychiatrist I asked two questions of myself about this book. Did I identify with the problems described here? And second, would I have found it valuable to read at an earlier stage in my career? The answer to both of these is a (qualified) yes.

The book is in two parts. The first consists of 16 interviews with eminent female psychiatrists and psychologists, which were almost certainly conducted by email. The questions are standardised, the responses polished and there is no in-depth probing of replies – which as a qualitative researcher I would have found more revealing. The second part of the book is a series of rather brief chapters which provide a helpful template for recognising the barriers and considering how to overcome them. These include putting yourself first, projecting confidence even if you don't feel it, being memorable, persistent and something I've found to be particularly important – networking.

The problems described are all too familiar – I've been subjected to mansplaining, *‘a man compelled to explain, especially to a woman, something that she already knows better than him.’* I've held back in conversation, fearful of asking questions. I've been the only woman in a committee room except for the person taking the minutes. Yet, all of these very talented women demonstrate how a combination of real passion for what you are interested in and the ambition to push yourself forwards, even when times are challenging, has resulted in outstanding academic success.

Almost all the interviewees are now at the pinnacle of their careers. Many came from similar professional families and more than a few acknowledge the role played by a supportive partner with the kind of job that made it easier to be both an academic clinician and a mother. There is no one still ‘finding their way’ that a younger me might have identified with and the majority work in biological psychiatry – the editors' own field – in the USA. I might have found it helpful to read their stories when I was younger, but on the other hand, I might have been more than a little intimidated too.

